# Sex Hormones Regulate *SHANK* Expression

**DOI:** 10.3389/fnmol.2018.00337

**Published:** 2018-09-25

**Authors:** Simone Berkel, Ahmed Eltokhi, Henning Fröhlich, Diana Porras-Gonzalez, Rafiullah Rafiullah, Rolf Sprengel, Gudrun A. Rappold

**Affiliations:** ^1^Department of Human Molecular Genetics, Institute of Human Genetics, Ruprecht-Karls-University, Heidelberg, Germany; ^2^Research Group of the Max Planck Institute for Medical Research at the Institute of Anatomy and Cell Biology, Ruprecht-Karls-University, Heidelberg, Germany

**Keywords:** autism spectrum disorders (ASD), sex differences, *SHANK*, SH-SY5Y cells, dihydrotestosterone, 17β-estradiol, androgen receptor knock-out mouse

## Abstract

Autism spectrum disorders (ASD) have a higher prevalence in male individuals compared to females, with a ratio of affected boys compared to girls of 4:1 for ASD and 11:1 for Asperger syndrome. Mutations in the *SHANK* genes (comprising *SHANK1*, *SHANK2* and *SHANK3*) coding for postsynaptic scaffolding proteins have been tightly associated with ASD. As early brain development is strongly influenced by sex hormones, we investigated the effect of dihydrotestosterone (DHT) and 17β-estradiol on *SHANK* expression in a human neuroblastoma cell model. Both sex hormones had a significant impact on the expression of all three *SHANK* genes, which could be effectively blocked by androgen and estrogen receptor antagonists. In neuron-specific androgen receptor knock-out mice (*Ar*^NesCre^), we found a nominal significant reduction of all *Shank* genes at postnatal day 7.5 in the cortex. In the developing cortex of wild-type (WT) CD1 mice, a sex-differential protein expression was identified for all Shanks at embryonic day 17.5 and postnatal day 7.5 with significantly higher protein levels in male compared to female mice. Together, we could show that *SHANK* expression is influenced by sex hormones leading to a sex-differential expression, thus providing novel insights into the sex bias in ASD.

## Introduction

Autism Spectrum disorders (ASD) are neurodevelopmental disorders characterized by restricted and repetitive behaviors, lack of imaginative play and impaired social interaction coupled with verbal and non-verbal communication deficits. Heritability is estimated between 70%–90%, and one out of 68 children in the United States is affected (Bailey et al., 1995; Developmental Disabilities Monitoring Network Surveillance Year 2010 Principal Investigators, Centers for Disease Control and Prevention (CDC), 2014). ASD symptoms persist throughout life and prenatal impairments develop into a postnatal manifestation (Rabaneda et al., 2014).

ASD occur more frequently in males compared to females with a ratio of 4:1 for ASD and 11:1 for Asperger syndrome (Gillberg et al., 2006; Fombonne, 2009; Werling, 2016); however, the mechanism behind this sex-differential risk is not well understood. A diagnostic bias may explain part of these differences as ASD are more visible and easier to be diagnosed in males (Bargiela et al., 2016). One hypothesis to explain the sex-differential risk postulates that ASD risk genes show sex-dimorphic expression as they may reside on the sex chromosomes and are influenced by skewed X chromosome inactivation or sex-specific imprinting defects on autosomes. Another hypothesis claims that ASD risk genes interact with sexually dimorphic pathways (Baron-Cohen et al., 2011; Werling et al., 2016). Prenatal and neonatal brain development is known to be influenced by sex hormones, suggesting that they may indeed play a role in the sex-differential expression of ASD symptoms (Werling, 2016). Estrogen enhances synaptogenesis and modulates synaptic plasticity and presumably has a neuroprotective effect (Beyer, 1999). Furthermore, a subset of adolescent ASD individuals presented elevated androgen levels (Tordjman et al., 1997) and individual differences in the development of typical autistic traits were shown to be influenced by fetal testosterone levels (Auyeung et al., 2009). To this end, a high testosterone level during development has been postulated as a risk factor for ASD (Baron-Cohen et al., 2011). Differences in synaptic composition and regional cerebral plasticity between the sexes were also proposed to account for the sex bias in ASD (Mottron et al., 2015).

SHANK proteins (SHANK1, SHANK2 and SHANK3) are scaffolding proteins at the postsynaptic site of excitatory synapses in the central nervous system (Kreienkamp, 2008), crucial for the formation, organization and signaling of excitatory synapses. *SHANK* gene variants have been associated with ASD, intellectual disability and schizophrenia (Leblond et al., 2014; Eltokhi et al., 2018).

To identify a putative regulatory role of sex hormones on *SHANK* expression, we first treated a human neuroblastoma cell line, SH-SY5Y, with dihydrotestosterone (DHT) or 17β-estradiol and quantified *SHANK* gene expression. In addition, *Shank* gene expression was evaluated pre- and postnatally in the cortex of mice lacking the androgen receptor (Ar) in neurons (*Ar*^NesCre^ mice). Moreover, we examined *Shank* gene and protein expression in the cortex of male and female wild-type (WT) CD1 mice at two different early developmental stages.

## Materials and Methods

### Animals

Mice were housed in the Interfacultary Biomedical Facility at the Heidelberg University (IBF), under a 12 h light-dark cycle and given *ad libitum* access to water and food. All procedures were conducted in strict compliance with the National Institutes of Health Guidelines for the Care and Use of Laboratory Animals and approved by the German Animal Welfare Act and performed according to the regulations of animal experimentation within Heidelberg University and the European Union (European Communities Council Directive 2010/63/EU (local license number: T-03/16, T-24/14)). CD1 (ICR) mice (Charles River) were used for sex-specific expression analyses at different developmental stages (*n* = 16 animals of each sex). The embryonic stage was calculated by vaginal plug check and by controlling the morphological parameters which accord to the respective Theiler stage. The day of birth was considered as postnatal day (P) 0.5. Sex genotyping was performed by PCR detection of the male-specific *Sry* gene on tail biopsies. Neuron-specific Ar knock-out mice (*Ar*^NesCre^) were generated by crossing female homozygous floxed Ar (*Arflox*) mice (*B6N.129-Ar*^tm1Verh^/Cnrm; De Gendt et al., 2004) with male Nestin-Cre deleter mice (*Tg*^(Nes-cre)1Kln^; MGI:2176173; Tronche et al., 1999) hemizygous for the floxed *Ar* allele. *Arflox* mice were obtained from the European Mouse Mutant Archive (EMMA, #02579) which had been backcrossed into the C57Bl/6N background for over 12 generations prior to the arrival at the IBF animal facility, as published previously (Fröhlich et al., 2017). Adult mice were sacrificed using CO_2_ and early postnatal animals were decapitated, then the frontal part of the cortex was dissected for further analysis.

Cre induced site-specific recombination was checked on cortical cDNA from E17.5 and P7.5 WT and Ar^NesCre^ brains by qPCR using a specific reverse primer residing in the floxed exon 2 to determine exon 2 deletion. Primer sequences are given in Supplementary Table S1.

### Cell Culture

Human neuroblastoma cells (SH-SY5Y) were grown on 75 cm flasks in Dulbecco’s modified Eagle medium (DMEM, Thermo Fisher Scientific), supplemented with 15% fetal calf serum, 1% non-essential amino acids and 1% Penicillin-streptomycin at 37°C in a humidified environment with 5% CO_2_. Cells were split at 80%–90% confluency and 8 × 10^5^ cells were plated per well on a 6-well cell culture plate in phenol red-free DMEM (Thermo Fisher Scientific) containing 1% charcoal dextran-treated calf serum for 24 h. Cells were treated either with 100 nM DHT (dissolved in methanol; Sigma-D-073-1ML), mock (100% methanol with the same dilution factor as DHT), 100 nM DHT combined with 1 μM flutamide (Sigma-F9397) or mock together with flutamide. To investigate the effect of 17β-estradiol, cells were treated either with 100 nM 17β-estradiol dissolved in 100% ethanol (Sigma-E8875), mock (100% ethanol with the same dilution factor as 17β-estradiol), 100 nM 17β-estradiol combined with 100 nM MPP (1,3-*Bis*(4-hydroxyphenyl)-4-methyl-5-[4-(2-piperidinylethoxy)phenol]-1*H*-pyrazole-dihydrochloride; Sigma-M7068), mock together with MPP, 100 nM 17β-estradiol plus 100 nM PHTPP (4-[2-Phenyl-5, 7-*bis*(trifluoromethyl)pyrazolo[1,5-*a*]pyrimidin-3-yl]phenol; Tocris-2662), mock plus PHTPP, 100 nM 17β-estradiol plus both MPP and PHTPP or mock plus both MPP and PHTPP. Cells were harvested after 4 h of treatment for mRNA and after 48 h for protein analyses.

### Quantitative Real Time PCR (qPCR)

Total RNA from SH-SY5Y cells (five experiments with six biological replicates for each condition), CD1 and *Ar*^NesCre^ mouse cortices at E17.5 and P7.5 was extracted with TRIzol (Invitrogen) according to the manufacturer’s instructions. Reverse transcription was performed using the SuperScript™ VILO™ cDNA Synthesis Kit (Invitrogen). Quantitative PCR was conducted using the SYBR Green Lo-Rox Fast Mix (Bioline) and the ABI 7,500 Fast Real-Time PCR system (Applied Biosystems). Each sample was analyzed in triplicates. Relative mRNA levels were assessed using the relative standard curve method by normalization to the following reference RNAs: ribosomal *18S RNA*, glyceraldehyde 3-phosphate dehydrogenase (*GAPDH*) mRNA, heat shock protein family D (HSP60) member1 (*HSPD1*) mRNA, succinate dehydrogenase complex subunit A mRNA (*SDHA*) and hypoxanthine phosphoribosyltransferase 1 mRNA (*HPRT1*). For the SH-SY5Y cell treatment with 17β-estradiol, only *18S*, *HSPD1* and *SDHA* were used as reference mRNAs, as *Gapdh* and *Hprt1* mRNA levels were influenced by estradiol (Schroder et al., 2009). Our analysis confirmed their differential expression in SH-SY5Y cells after 17β-estradiol treatment when normalized to *18S* (Supplementary Table S2A). The relative expression values for mock treatment were set to 1. Calculated values are presented as normalized relative expression ratios. The sequences for the oligonucleotides used are given in Supplementary Table S1.

### Protein Analysis

Immunefluorescence microscopy was carried out on fixed SH-SY5Y cells using the primary antibodies anti-Ar (Abcam, ab74272, 1:100 dilution) and anti-estrogen receptor α (Abcam, ab661002, 1:100 dilution), and as secondary antibodies Alexa fluor 488 goat anti-rabbit or Alexa fluor 488 goat anti-mouse (Thermo Fisher Scientific, 1:1,000 dilution).

Protein extraction from SH-SY5Y cells and from mouse frontal cortex (using the Polytron PT1200E, Kinematica AG) was performed at 4°C using RIPA buffer supplemented with SIGMAFAST protease inhibitor (S8820; Sigma). Protein concentrations were determined with the BCA protein assay kit (Pierce). Western blot analysis was executed using the Odyssey Infrared Imaging System (LI-COR Biosciences). Twenty microgram of proteins were separated on Novex WedgeWell 4%–12% Tris Glycine Gels (Thermo Fisher Scientific) and transferred to PVDF membrane (Millipore). PVDF membranes were probed with mouse anti-pan-SHANK (1:500; Neuromab), mouse monoclonal β3-tubulin (1:20,000; Promega-G7121), anti-SHANK1 (Synaptic Systems, polyclonal rabbit purified antibody, 1:500 dilution), anti-SHANK2 (Synaptic Systems, polyclonal guinea pig antiserum, 1:500 dilution), anti-SHANK3 (ab140030, Abcam, 1:1,000 dilution). IRDye 800CW donkey anti-mouse, IRDye 680LT donkey anti-guinea pig or IRDye 680RD donkey anti-rabbit (1:15,000 dilution; LI-COR Biosciences) immuno-positive signals were quantified using the Image Studio Lite 3.1 software (LI-COR Biosciences). The Page Ruler Prestained Protein ladder (10–180 kDa) and the Spectra Multicolor High Range Protein ladder (40–300 kDa; Thermo Fisher Scientific) were used as protein size marker. Shank expression was normalized to the amount of β3-tubulin and the values obtained for the male cortices were set to 1.

### nCounter Analysis

Total RNA from conditional *Ar*^NesCre^ mouse brain cortex was extracted with TRIzol (Invitrogen) and the gene expression profile was investigated by nCounter expression analysis at the nCounter Core Facility Heidelberg, using the nCounter Dx analysis system GEN1 (NanoString Technologies). A customized Elements codeset with seven target genes and four reference genes was applied. (For probe design see Supplementary Table S3). The detailed workflow is described at https://www.nanostring.com/support/product-support/support-workflow. Background correction and normalization of data were performed using the nSolver Analysis Software 3.0 (NanoString Technologies). A positive control and reference gene normalization was performed according to the Gene expression analysis guideline from NanoString Technologies^1^. The most stable expressed genes *Gapdh*, *Hspd1*, *Sdha* and *Hprt1* were selected for normalization based on the geNorm method (Vandesompele et al., 2002). The unit of measurement is given in “codeset counts” and the codeset counts of the WT animals were set to 100%. The absolute numbers are provided in Supplementary Table S4.

### Statistical Analysis

IBM SPSS STATISTICS 21, Prism 6 software (GraphPad Software) and Microsoft Office Excel software were used for data analysis. Two-way ANOVA was performed for comparing RNA expression levels between hormone-treated and mock-treated SH-SY5Y cells with treatment and experiment as the two factors. To compare gene expression levels between male and female cortices, two-way ANOVA was used with litter and sex as influencing factors. According to Bonferroni correction for multiple testing, a *P*-value threshold of ≤ 0.01 was considered significant (*n* = 5 different tests). For the expression analysis in the conditional *Ar*^NesCre^ mouse to compare differences between the WT and the knock-out mice and for the quantification of the Shank protein in male and female cortices in the western blot experiments, an unpaired two-tailed Student’s *t*-test was used, with a *P*-value of ≤ 0.05 considered as nominal significant. All data are presented as mean values ± standard error of the mean (SEM).

## Results

### *SHANK* Expression in Human Cells Treated With Dihydrotestosterone and 17β-Estradiol

To receive a first indication whether androgen has an effect on *SHANK* gene expression, we employed a human neuroblastoma cell model (SH-SY5Y). SH-SY5Y cells express all three SHANKs, as well as the androgen, estrogen α and β receptors (AR, ERα and ERβ; Chamniansawat and Chongthammakun, 2009, 2010; Sarachana et al., 2011; Grassi et al., 2013; Sarachana and Hu, 2013). The expression of AR and ERα in the SH-SY5Y cells is shown in Supplementary Figure S1A. After stimulation with DHT for 2 h, the overall expression of AR increased in the cells. Stimulation with 17 β-estradiol did not increase the expression level of ERα, but more protein was found in the nucleus (Supplementary Figure S1A).

To determine a regulatory influence on *SHANK* gene expression, we treated SH-SY5Y cells with the androgen DHT at different concentrations (1 nM, 10 nM and 100 nM; Supplementary Figure S1B). Based on the literature, DHT concentrations between 1 nM and 100 nM are considered to be within the physiological range and have been used in similar studies (Sarachana et al., 2011; Grassi et al., 2013). The strongest effects on *SHANK* expression were obtained by treatment with 100 nM for 4 h. *SHANK* gene expression was measured by qPCR and normalized to five androgen-independent reference RNAs: the ribosomal *18S* RNA, the mRNAs for glyceraldehyde 3-phosphate dehydrogenase (*GAPDH*), the heat shock protein family D (HSP60) member1 (*HSPD1*), the succinate dehydrogenase complex subunit A mRNA (*SDHA*) and hypoxanthine phosphoribosyltransferase 1 mRNA (*HPRT1*). The androgen independance of the reference genes was shown by the unchanged levels of *GAPDH*, *HPRT1*, *HSPD1* and *SDHA* normalized to *18S* in DHT and mock-treated SH-SY5Y cells (Supplementary Table S2A). *PSD95*, a gene known to be regulated by sex hormones (Akama and McEwen, 2003; Liu et al., 2008), and the X-chromosomal gene *MECP2*, which shows a transient sex-specific expression difference in the developing rat brain (Kurian et al., 2007), were included as additional markers in the expression analysis.

**Figure 1:**
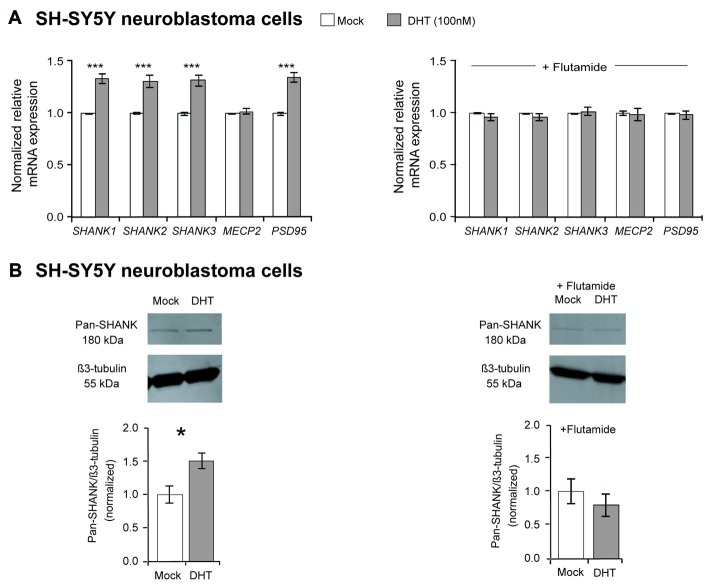
Effect of dihydrotestosterone (DHT) on *SHANK* gene expression in SH-SY5Y cells. **(A)** Quantification of *SHANK*, *MECP2* and *PSD95* gene expression by qPCR after 4 h of treatment with either 100 nM DHT or a combination of 100 nM DHT and 1 μM flutamide (*n* = 5 experiments with six biological replicates for each condition). Gene expression was normalized against five reference genes (*18S*, *GAPDH*, *HPRT1*, *HSPD1* and *SDHA*; two-way ANOVA, ***P ≤ 0.001, vs. mock). Bonferroni threshold: *n* = 5 tests, *P* ≤ 0.01. **(B)** An increase of SHANK protein levels by 50% was identified after 48 h of 100 nM DHT treatment by western blot analysis, whereas no difference was determined after 48 h of treatment with DHT combined with flutamide (*n* = 5 experiments, unpaired two-tailed Student’s *t*-tests, *P ≤ 0.05). Error bars indicate standard error of the mean (SEM).

Our mRNA expression analysis showed that the DHT treatment significantly elevated the expression of all three *SHANK* genes by about 35% (*P*-values ≤ 0.001), compared to mock-treated SH-SY5Y cells (Figure 1A). Expression levels of *MECP2* were not influenced, whereas *PSD95* expression was significantly increased after DHT treatment (Figure 1A). The regulatory effect of DHT on *SHANK* gene expression could also be detected by an increased SHANK immuno-signal in western blots using an anti-pan-SHANK antibody and an anti-SHANK3 antibody (Figure 1B, Supplementary Figures S1C,D). When the SH-SY5Y cells were treated with DHT combined with the anti-androgen flutamide, the regulatory effect of DHT on *SHANK* and *PSD95* mRNA levels was completely abolished and as shown with a pan-Shank antibody also on the protein level, demonstrating that the increased *SHANK* and *PSD95* levels after DHT treatment are mediated by the stimulation of the AR (Figures 1A,B).

**Figure 2:**
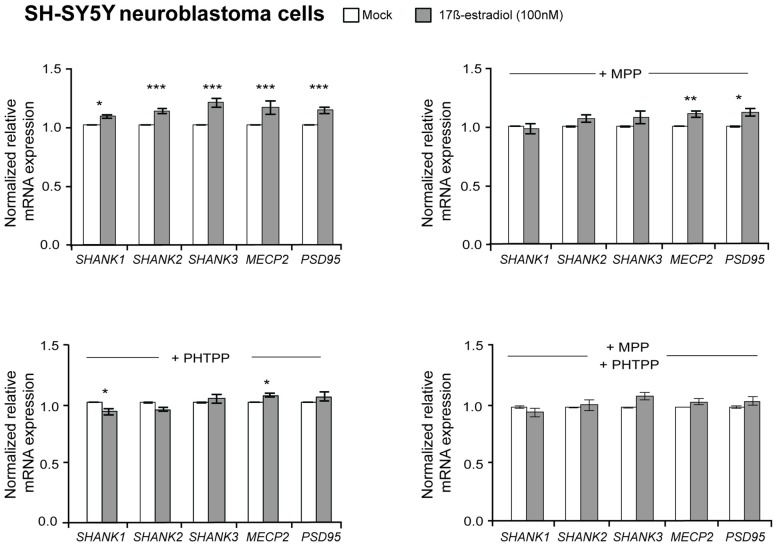
Effect of 17β-estradiol on *SHANK* gene expression in SH-SY5Y cells. Gene expression analysis of *SHANK, MECP2* and *PSD95* after 4 h of treatment with 100 nM 17β-estradiol. The expression was also analyzed after stimulation with 100 nM 17β-estradiol in combination with a selective blocking of estrogen receptor α (100 nM MPP), estrogen receptor β (100 nM PHTPP) or a combination of both (*n* = 5 experiments with six biological replicates for each condition). Gene expression was normalized against three reference genes (*18S*, *HSPD1* and *SDHA*). Error bars indicate SEM (two-way ANOVA, *P ≤ 0.05, **P ≤ 0.01, ***P ≤ 0.001, vs. mock-treatment control). Bonferroni correction: *n* = 5 tests, *P* ≤ 0.01.

To elucidate if estrogens also regulate *SHANK* gene expression, 100 nM 17β-estradiol was used to stimulate the estrogen receptors in SH-SY5Y cells. After 17β-estradiol treatment for 4 h, a minor enhancement of *SHANK, MECP2* and *PSD95* expression could be observed. Now we restricted the normalization against reference gene expression of *18S*, *HSPD1* and *SDHA*, since *GAPDH* and *HPRT1* expression was affected by 17β-estradiol (Supplementary Table S2A). Our quantification revealed an increase of 15% mRNA expression for *SHANK*, *PSD95* and *MECP2*, when compared to mock-treated SH-SY5Y cells (*P*-value for *SHANK1* = 0.02; *P*-values for *SHANK2*, *SHANK3*, *MECP2* and *PSD95* ≤ 0.001; Figure 2). However, a regulatory influence on SHANK protein expression could not be detected in immunoblots (Supplementary Figure S1E). By blocking the ERα or ERβ receptor subtypes with the selective ERα antagonist MPP or ERβ antagonist PHTPP, the effect of 17β-estradiol on *SHANK* expression by MPP was gone, whereas the blocking of ERβ with the antagonist PHTPP abrogated the effect on *SHANK* and *PSD95* expression (Figure 2). The combined blocking of both ERs antagonized the 17β-estradiol-stimulated expression of *SHANK*, *PSD95* and *MECP2* (Figure 2). Thus, besides AR, also ERα and ERβ signaling contributes to the expression of *SHANK* genes in the human SH-SY5Y cell line.

### Expression Analysis of *Shank* Genes in Neuron-Specific Conditional Androgen Receptor Knock-out Mice (*Ar*^NesCre^)

**Figure 3:**
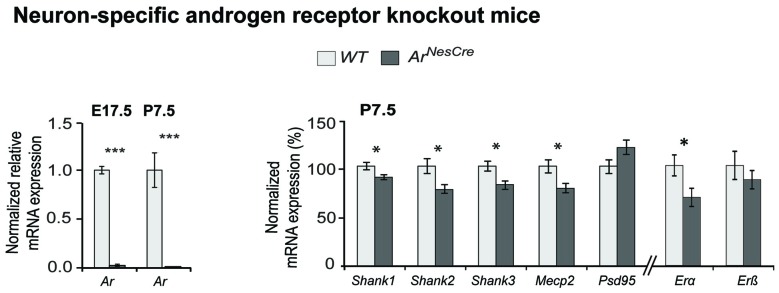
Comparative *Shank* gene expression analysis by nCounter in the cortex of wild-type (WT) and *Ar*^NesCre^ mice. (Left) Loss of androgen receptor (*Ar*) expression in cortical neurons was confirmed by qPCR. The deletion of *Ar*-exon2 was shown on mRNA level in cortical tissue. (Right) Gene expression analysis in cortex of WT and *Ar*^NesCre^ mice at P7.5. The loss of Ar resulted in a decreased expression of the *Shank* and *Mecp2* genes (*n* = 7 WT and seven KO mice, two male and five female animals in each group). The analysis could not be stratified by sex due to low numbers. Gene expression was normalized against four reference genes (*Gapdh*, *Hprt1*, *Hspd1* and *Sdha*). Error bars indicate SEM (unpaired two-tailed Student’s *t*-tests, *P ≤ 0.05; ***P ≤ 0.001).

To provide *in vivo* evidence for the Ar regulatory influence on *Shank* gene expression during neurodevelopment, we used a neuron-specific Ar knock-out mouse (*Ar*^NesCre^; Fröhlich et al., 2017). We analyzed *Shank* mRNA expression in the cortex of *Ar*^NesCre^ mice at two developmental stages, E17.5 and P7.5 (Figure 3, Supplementary Figure S2A). In this critical period of brain development, sex differences in the spatiotemporal expression of *Ar*, *Erα* and *Erβ* in mouse brain as well as differences in testosterone levels were previously reported (Mogi et al., 2015). Furthermore, it is known that in the human cortex, size and sex hormone levels differ between males and females (Lombardo et al., 2012; Lai et al., 2013). By nCounter analysis, we identified a reduced mRNA expression of all three *Shanks* and* Mecp2* in the *Ar*^NesCre^ mice compared to WT at P7.5 with nominal significance (Figure 3), supporting the contribution of Ar signaling in the specific regulation of the *Shank* gene expression during development.

### Expression Analysis of the *Shank* Genes and Proteins in the Male and Female Mouse Cortex

**Figure 4:**
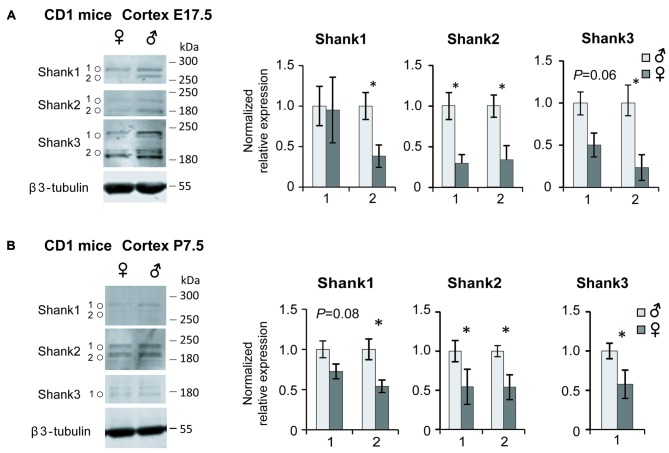
Shank protein analysis in the male and female mouse cortex. Levels of Shank1, Shank2 and Shank3 protein in the frontal cortex of male and female at E17.5 **(A)** and P7.5 **(B)**; (*n* = 4 of each sex), *P < 0.05, unpaired two-tailed Student’s *t*-tests. Isoform expression differences were observed between the two developmental stages for Shank3.

Further support for a sex-specific regulation of *Shank* expression could be provided by comparing the expression levels in the frontal cortices of male and female CD1 WT mice at two different developmental stages, E17.5 and P7.5. The sex-independent expression of the reference genes was confirmed by showing equal levels of *Gapdh*, *Sdha*, *Hprt1* and *Hspd1* expression, normalized to *18s*, in male and female mice cortices (Supplementary Table S2B). In the comparative qPCR analysis, significantly elevated *Shank1*, *Shank3* and *Mecp2* expression levels could be detected in the cortices of female compared to the male mice at E17.5 (Supplementary Figure S2B). At P7.5, our analysis revealed elevated *Shank1* mRNA expression in the female cortex with nominal significance, as well as significantly elevated *Psd95* expression (Supplementary Figure S2B). Then we analyzed Shank protein expression in whole protein lysate from the frontal cortex of male and female mice at E17.5 and P7.5 by western blot. In contrast to the mRNA expression analysis, we found significantly higher expression levels for all three Shank proteins in the male compared to female cortices at both developmental stages. Shank expression is decreased in females by 50%–77% at E17.5 (Figure 4A) and by 43%–47% at P7.5 (Figure 4B), indicating that Shank expression differences are more pronounced at the earlier stage of E17.5.

## Discussion

Males show higher prenatal and postnatal testosterone levels than females (Baron-Cohen, 2002; Werling, 2016). Elevated prenatal exposure to testosterone and /or irregular steroidogenic activity have been postulated to be associated with elevated ASD risk (Auyeung et al., 2009). Testosterone is metabolized in the brain into DHT or converted into estradiol by the enzyme aromatase (CYP19A1). Therefore, we have investigated the influence of both hormones on the expression of the *SHANK* genes, which are strongly linked to ASD pathology (Leblond et al., 2014). We could show in the SH-SY5Y neuroblastoma cell line, a widely used neuronal cell model to investigate hormonal influences of androgens and estrogens (Sarachana et al., 2011; Grassi et al., 2013; Nakaso et al., 2014; Sun et al., 2017), that the effect of DHT and 17β-estradiol on the expression of all three Shanks could be effectively blocked by androgen and estrogen receptor antagonists. *SHANK* gene expression was shown to be regulated by DHT, directly modulated by AR signaling. DHT increased *SHANK* mRNA levels by 35% and 17β-estradiol increased *SHANK* mRNA levels by 15%, suggesting a role as transcriptional fine-tuners. Protein levels were found to be increased by DHT treatment by 50%, indicating that the major effect is seen at the level of translation.

More direct evidence for the contribution of AR signaling on the regulation of *SHANK* gene expression could be demonstrated by the analysis of mice with *Ar* gene deletion in neurons (ArNesCre). *Ar*^NesCre^ mice show reduced sexual, territorial and aggressive behavior (Raskin et al., 2009; Studer et al., 2015). Male *Ar*^NesCre^ mice lack social memory when presented to male conspecifics but do not show ASD-like impairments in social interactions with other mice (Karlsson et al., 2016). We found that in absence of the Ar in neurons, the expression of all three *Shanks* was reduced and reached nominal significance, supporting the role of Ar activity in the regulation of *Shank* expression during brain development. Previous data has shown that *Ar* and *Shank* genes have overlapping expression patterns in the cortex, striatum and hippocampus (Lein et al., 2007; Fröhlich et al., 2017; Monteiro and Feng, 2017). Androgen- and estrogen-dependent gene regulation has also been described for genes involved in the function of glutamatergic synapses in the medial preoptic area and the ventromedial hypothalamus of rats, after exposure to anti-androgenic and/or estrogenic treatment or a combination of both *in utero* (Lichtensteiger et al., 2015). In that study, *Shank1* and *Shank2* expression was altered in young male rats and *Shank2* and *Shank3* in female animals at P6, hereby providing additional *in vivo* evidence of a sex hormone regulation of *Shank* gene expression in specific brain regions.

Palindromic and dihexameric motifs of androgen responsive elements were described in the promoters or enhancers of all three *SHANK* genes: half and full sites in *SHANK2* and half sites in *SHANK1* and *SHANK3* (Wilson et al., 2016). An AR-binding site was also recently identified in an intron and in the distant promoter region of *SHANK2* by ChIP-Seq (Quartier et al., 2018). These results point to a direct regulatory influence of DHT on *SHANK2*. In a different study, direct and indirect interaction partners of SHANKs, such as PSD-95 and the AMPA receptor subunits GluA1 and GluA2, were reported to be androgen-responsive (Trabzuni et al., 2013), suggesting that synergistic effects at the synapse between multiple proteins strengthen the individual effects.

Despite the fact that *Shank1* mRNA expression levels are lower in male mice at P7.5, we could show that protein levels of all Shanks are higher in males during late prenatal and early postnatal cortical development in WT mice (E17.5 and P7.5). The RNA level does not always correlate with protein levels, especially for proteins with a long half-life or in cases of negative feed-back when the increase in protein level decreases the expression of RNA. In a previous study on genome-wide scale, it was also found that the cellular abundance of protein is predominantly controlled at the level of translation (Schwanhausser et al., 2011). Between E17.5 and P7.5, *Ar* expression and testosterone level differ between male and female mice (Mogi et al., 2015). Male mice have about three times higher blood testosterone levels than female embryos at E17 (Vom Saal, 1983) and show significantly higher testosterone levels in the brain (ICR/CD1 strain) at E19 (Mogi et al., 2015). The finding that DHT increases *SHANK* mRNA and protein expression in SH-SY5Y cells, suggests that high levels of testosterone in the male brain may result in elevated SHANK protein expression. The discrepancy between high protein and low mRNA levels in CD1 mice might be caused by negative feedback mechanisms, suggesting that high Shank protein levels lead to lower Shank mRNA levels. However, SH-SY5Y cells may not entirely reflect the regulatory potential of testosterone signaling in cortical neurons, which are a heterogeneous population of cells that may respond individually differently to DHT. Therefore, the data obtained from established cell lines need to be verified in the living mouse.

In mice, the perinatal developmental period between E17 and P7.5 might be a vulnerable time window for *Shank* expression when sex differences in the cortex might intersect with ASD etiological pathways. Physiologically higher SHANK protein expression levels in males compared to females implicate that genetic variants may have a higher penetrance in males, leading to a larger proportion of males with a diagnosis of ASD. Clinical studies reported elevated androgen levels in ASD-affected individuals (Baron-Cohen, 2002; Werling, 2016). Our results imply that Shank expression may be increased in these individuals, at least during the developmental vulnerable stages. In contrast, most ASD individuals have been identified with *SHANK* deletions and various point mutations, indicating that SHANK levels are very likely reduced in these ASD individuals. Nevertheless, it cannot be excluded that a general dysregulation of SHANK gene expression contributes to ASD pathology, as *SHANK3* gene duplications have also been identified in individuals with Asperger syndrome (Durand et al., 2007).

*SHANK* genes reside on autosomes, and *SHANK* variants are randomly distributed across male and female subjects. So far, only copy number variant (CNV) deletions encompassing the *SHANK1* gene segregated in male carriers with high-functioning autism and showed a clearly reduced penetrance in female individuals (Sato et al., 2012) and some *SHANK* variants have been found in male autistic patients that were inherited from healthy mothers (for a recent review see (Eltokhi et al., 2018)).

Sex differences in gene expression were previously studied in prenatal and adult neocortical tissue of human post-mortem brain (Trabzuni et al., 2013; Werling et al., 2016). Only a small proportion of the analyzed genes, however, were found to be expressed in a sex-specific manner (96 prenatal, 58 adult; Werling et al., 2016). No evidence was obtained for sex-differential expression of the *SHANKs* or other ASD-associated genes like *RORA* and *FOXP1* (Sarachana et al., 2011; Takayama et al., 2014; Fröhlich et al., 2017). However, in those studies, the analyzed cortical samples were from prenatal (16–22 weeks) or adult samples and did not match with the investigated developmental stages used in our study, suggesting that the days shortly before or after birth are sensitive for sex hormone-regulated SHANK/Shank expression.

Together, we found that the expression of the ASD-associated *SHANK* gene family is upregulated by DHT in SH-SY5Y cells and that in mice all three Shanks showed elevated protein expression levels in males during late prenatal and early postnatal cortical development, that most likely are facilitated by high testosterone levels of males during these developmental time windows. Our results provide novel insights in the understanding of the sex bias in ASD.

## Author Contributions

GR and SB designed the study. SB performed the mouse experiments and protein analysis. AE performed the cell culture experiments and mRNA expression analysis. HF and RR generated the *Ar*^NesCre^ mouse line and provided tissue. DP-G contributed to the cell culture and mouse experiments. SB and AE performed the data analysis. SB, AE, GR, HF and RS contributed to the data interpretation. SB, AE and GR wrote the manuscript. All authors contributed to and reviewed the final manuscript.

## Conflict of Interest Statement

The authors declare that the research was conducted in the absence of any commercial or financial relationships that could be construed as a potential conflict of interest.

## Footnotes

^1^https://www.nanostring.com/application/files/7715/1251/5220/Gene_Expression_Data_Analysis_Guidelines.pdf; accessed June 2018
