# Supplementary material for: Sex Hormones Regulate SHANK Expression
Source: Front Mol Neurosci. 2018 Sep 25;11:337. doi: 10.3389/fnmol.2018.00337 (PMC6167484; doi:10.3389/fnmol.2018.00337)
Supplement: Supplementary file 1 [file Data_Sheet_1.pdf]

frontiers

Supplementary Material

Sex hormones regulate *SHANK* gene expression

Simone Berkel[1]*, Ahmed Eltokhi[1,2]*, Henning Fröhlich[1], Diana Porras Gonzalez[1], Rafiullah Rafiullah[1], Rolf Sprengel[2], Gudrun A. Rappold[1]

* Authors contributed equally to this work

Correspondence: Prof. Dr. Gudrun Rappold: Gudrun.Rappold@med.uni-heidelberg.de

Supplementary Figures and Tables

1. Supplementary Figures

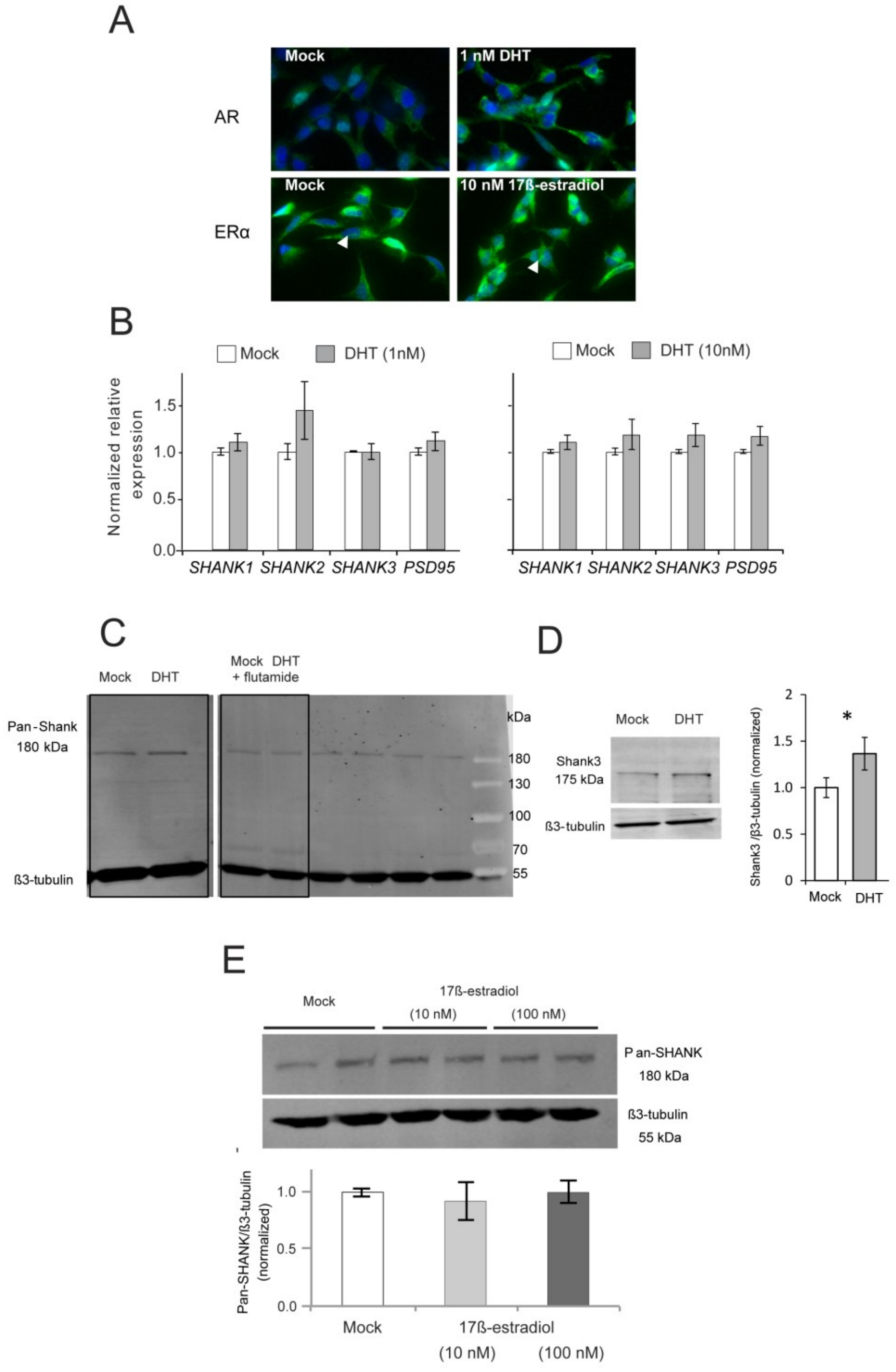

Supplementary Figure S1. Effect of DHT and 17β-estradiol treatments in SH-SY5Y cells

(A) Immunofluorescence staining showing the effect of 1 nM DHT and 10 nM 17ß-estradiol treatments for 2 hr in SH-SY5Y cells on AR and ERα expression, respectively. The expression of AR was increased after the DHT treatment. The expression of ERα was localized in the nucleus after the 17ß-estradiol treatment without an increase in the overall expression. (B) Gene expression analysis of *SHANK1-3* after 4 hrs of treatment with 1 nM DHT (n= 3 experiments with 6 biological replicates for each condition), and 10 nM DHT (n= 4 experiments with 6 biological replicates for each condition) (2-way ANOVA, versus mock). *PSD95* was used as a positive control. Gene expression was normalized against *18S*. (C) Full western blot pictures showing a band with a size of 180 kDa using Pan-Shank and a band with a size of 55 kDa using ß3-tubulin. The marked frames indicate the corresponding bands in Figure 1B. (D) An increase of Shank3 protein level by 40% was identified after 48 hours of 100 nM DHT treatment (n= 3, unpaired two-tailed Student's t-tests, * $P \leq 0.05$) by western blot analysis. (E) Western blot analysis did not show any significant effect of 17ß-estradiol treatment on SHANK protein expression in SH-SY5Y cells (10 nM, 100 nM 17ß-estradiol, n= 3 per treatment group). Error bars indicate SEM.

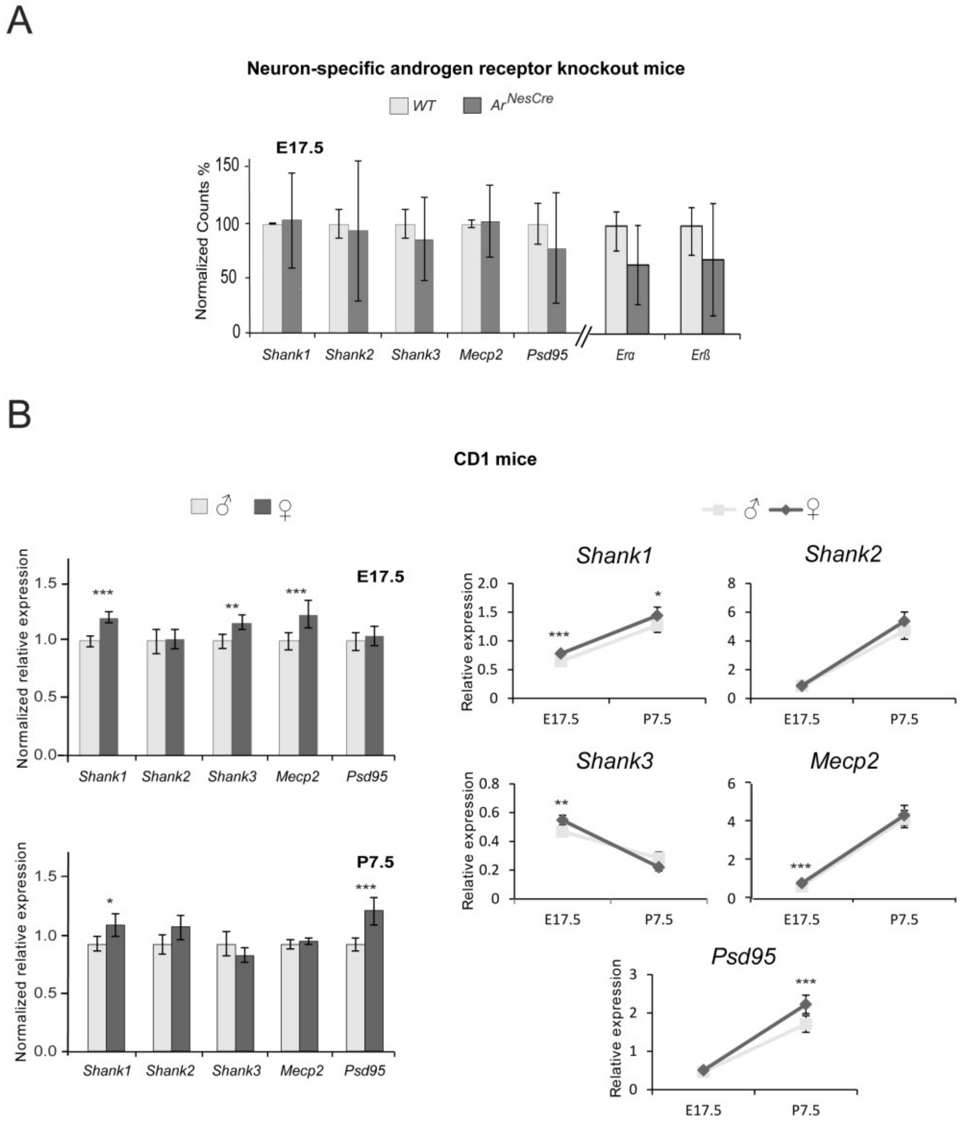

Supplementary Figure S2. *Shank* gene expression analysis in the male and female mouse cortex (A) Gene expression analysis in the cortex of wild-type (C57Bl/6N) and neuron-specific androgen receptor knock-out mice at E17.5 (n= 6 *WT* and 6 *Ar*^{NesCre} mice, 3 male and 3 female animals in each group). The expression analyses were normalized against *Gapdh*, *Hprt1*, *Hspd1* and *Sdha*. Error bars indicate SEM. (B) qPCR analysis revealed gene expression differences in the male and female mouse cortex at E17.5 for *Shank1* (*P*=0.001), *Shank3 (P*= 0.007) and *Mecp2* (*P*=0.0005) (n= 16 male,16 female). At developmental stage P7.5, a sex-differential expression was obtained for *Psd95* (*P*= 0.001) and *Shank1* (*P*= 0.03) (n=18 male, 17 female). *Shank1*, *Shank2*, *Mecp2* and *Psd95* expression

increased in the cortex from E17.5 to P7.5. For *Shank3*, expression decreased at P7.5 in both male and female mice. The respective gene expression was normalized against five reference genes (*18s*, *Gapdh*, *Hprt1*, *Hspd1* and *Sdha*). Error bars indicate SEM (2-way ANOVA, * $P \leq 0.05$, ** $P \leq 0.01$, *** $P \leq 0.001$). Bonferroni correction: n= 5 tests for each developmental stage, $P \leq 0.01$.

2. Supplementary Tables

Amplicon	Species	Primer Name	Sequence 5´ to 3´
18S	human	18S_F	GATGGGCGGCGGAAAATAG
	human	18S_R	GCGTGGATTCTGCATAATGGT
GAPDH	human	GAPDH_F	CTGGGCTACACTGAGCACC
	human	GAPDH_R	AAGTGGTCGTTGAGGGCAATG
HPRT1	human	HPRT1_Ex3-5_F	TGATAGATCCATTCCTATGACTGTAGA
	human	HPRT1_Ex3-5_R	AAGACATTCTTTCCAGTTAAAGTTGAG
HSPD1	human	HSPD1_F	GATGGAGAAGCTCTAAGTACACT
	human	HSPD1_R	GCTGGTTCTTTCTATTGTCACCA
SDHA	human	SDHA-Ex2-3 F	TGGGAACAAGAGGGCATCTG
	human	SDHA-Ex2-3 R	CCACCACTGCATCAAATTCATG
SHANK1	human	SHANK1_F	CTCCCTGCGTTCCAAATCTA
	human	SHANK1_R	GGCTGCTGCTCGTACTCC
SHANK2	human	SHANK2_F	CTTTGGATTCGTGCTTCGAG
	human	SHANK2_R	CATCCACGGACTCCAGGTA
SHANK3	human	SHANK3_F	TTCCACGGACCAAGTCTGTA
	human	SHANK3_R	GTCTTGCATCGAGGTGCTC
MECP2	human	MECP2_F	GTGGAGTTGATTGCGTACTTCG
	human	MECP2_R	CCCTCTCCCAGTTACCGTGAA
PSD95	human	PSD95_F	TCACAACCTCTTATTCCCAGCA
	human	PSD95_R	CATGGCTGTGGGGTAGTAGTCG
18s	mouse	18s_F	CTTAGAGGGACAAGTGGCG
	mouse	18s_R	ACGCTGAGCCAGTCAGTGTA
Gapdh	mouse	Gapdh_F	ACAGTCCATGCCATCACTGCC
	mouse	Gapdh_R	GCCTGCTTCACCACCTTCTTG
Hprt1	mouse	Hprt1_F	TCCTCCTCAGACCGCTTTT
	mouse	Hprt1_R	CCTGGTTCATCATCGCTAATC
Hspd1	mouse	Hspd1_F	AGCTGTTACAATGGGGCCAA
	mouse	Hspd1_R	ATCCCCAGCCTCTTCGTTTG
Sdha	mouse	Sdha_F	CATGCCAGGGAAGATTACAAA
	mouse	Sdha_R	GTTCCCCAAACGGCTTCT
Shank1	mouse	mShank1_F	GTCCTACCAGGCGCAAGGCG
	mouse	mShank1_R	CCCAGAAGCCTCCTTCCCCGA
Shank2	mouse	mShank2_F	TGCTGCCAGTGACTGCATTATTGA
	mouse	mShank2_R	CAGGGCTGGAAATGCTGGCGT
Shank3	mouse	mShank3_F	AGGGAACCGTGAAGGGCCGA
	mouse	mShank3_R	TGGCGGAAGAGACGCTTCGT
Mecp2	mouse	Mecp2_F	GCGAGGAGGAGAGACTGGA
	mouse	Mecp2_R	TTCTTGTCTTTCTTCGCCTTCT
Psd95	mouse	Psd95_F	CTCTGCGAAGCAACCCCAA
	mouse	Psd95_R	TCTTCGTCGCTGGCGTCAATTA
Ar-knockout	mouse	mAr_Ex1F	GCCTCCGAAGTGTGGTATCC
	mouse	mAr_Ex2R	TGGTCCCTGGTACTGTCCAA

Supplementary Table S1. qPCR primer list

A

	GAPDH	*HPRT1*	*HSPD1*	*SDHA*
**DHT versus Mock**	0.202	0.335	0.316	0.194
**17ß-estradiol versus Mock**	0.097	0.032	0.229	0.64

B

	Gapdh	*Hprt1*	*Hspd1*	*Sdha*
**E17.5**	0.2055	0.8219	0.2179	0.9228
**P7.5**	0.7953	0.1236	0.6343	0.9668

Supplementary Table S2. (A) Analysis of the influence of DHT and 17ß-estradiol treatment on reference gene expression. 2-way ANOVA *P*-values are shown. The analyzed reference genes were normalized against *18S*. As *HPRT1* and *GAPDH* expression is influenced by 17ß-estradiol they were excluded from 17ß-estradiol treatment analysis. (B) Quantification of the relative reference gene expression during different developmental stages. 2- way ANOVA *P*-values are shown. The analyzed reference genes were normalized against *18S*.

Gene	Reference	Probe	Sequence
Gapdh	NM_008084.2	A	ATGGGCTTCCCGTTGATGACAAGCTTCCCATTCTCCCTCAAGACCTAAGCGACAGCGTGACCTTGTTTCA
Hspd1	NM_010477.4	A	CACAGTTCTTCCCTTTGGCCCCATTGTAACAGCTACAGCATCTGCTAAAACATCCTCTTCTTTTCTTGGTGTTGAGAAGATGCTC
Sdha	NM_023281.1	A	GTAATCATACTCATCGACCCGCACTTTGTAATCTTCCCTGGCATGGGCTCCACAATTCTGCGGGTTAGCAGGAAGGTTAGGGAAC
Hprt1	NM_013556.2	A	AACAAATCTAGGTCATAACCTGGTTCATCATCGCTAATCACGACGCTGGGCTGTTGAGATTATTGAGCTTCATCATGACCAGAAG
Shank1	NM_001034115.1	A	TCACCGCCATGAAGGAGCGACCGGGTACCGCTGAATAGAGCTTCCTACGTCTTTCGGGTTATATCTATCATTTACTTGACACCCT
Shank2	NM_001113373.2	A	TCCACGGACTCCAGGTACTGCAGGGCTGGAAATGCTGGCGTGGGTGTGAACAACAGCCACTTTTTTTCCAAATTTTGCAAGAGCC
Shank3	NM_021423.3	A	CTTCGTCCGGTCCTCTCTGGTTTCATGCCGGGTGTCATACTGTCGCACCGTGTGGACGGCAACTCAGAGATAACGCATAT
Mecp2	NM_010788.2	A	TGAACACCTTCTGATGCTGCTGCCTTTGGTCTCCCAGCCTGGAGTTTATGTATTGCCAACGAGTTTGTCTTT
Psd95	NM_007864.3	A	GGCCTGGCTCAAGAAACCGCAGTCCTTGGTCTTGTCGTAGTCAAACAAACTGGAGAGAGAAGTGAAGACGATTTAACCCA
Gapdh	NM_008084.2	B	CGAAAGCCATGACCTCCGATCACTCATTTGATGTTAGTGGGGTCTCGCTCCTGGAAGATGGTG
Hspd1	NM_010477.4	B	CGAAAGCCATGACCTCCGATCACTCGTGACCCCATCTTTTGTTACTTTGGGACTTCCCCAACTCTGTTCAATAAT
Sdha	NM_023281.1	B	CGAAAGCCATGACCTCCGATCACTCCAGTGTTCCCCAAACGGCTTCTTCTGCTGTCCCTGGATGGGCTTGGA
Hprt1	NM_013556.2	B	CGAAAGCCATGACCTCCGATCACTCGAGGAATAAACACTTTTTCCAAATCCTCGGCATAATGATTAGGTATACAA
Shank1	NM_001034115.1	B	CGAAAGCCATGACCTCCGATCACTCCTTTACTCAGAGAGATCTCCCCCTCGCCTTGCGCCTGGTAGGACT
Shank2	NM_001113373.2	B	CGAAAGCCATGACCTCCGATCACTCTCCTTAGTCCGGCTTGCCATGCCACCCCACCTTCA
Shank3	NM_021423.3	B	CGAAAGCCATGACCTCCGATCACTCTGTGAAGTGAGGCTGTCATAGGAACCCACAGTGTAGTGGCGGAAGAGACG
Mecp2	NM_010788.2	B	CGAAAGCCATGACCTCCGATCACTCGCATCTTGACAACAAGTTTCCCAGGGCTCTTCTCCAGGACCCTTTTCACC
Psd95	NM_007864.3	B	CGAAAGCCATGACCTCCGATCACTCTCGTCGCTGGCGTCAATTACATGAAGCACATCCCCAAAGTGGAAGCTCAG

Supplementary Table S3. Sequences of nCounter probes

A

E17.5

	Shank1	*Shank2*	*Shank3*	*Mecp2*	*Psd95*	*Er1*	*Er2*
Ar^{NesCre}	8603.28	6229.19	3063.5	7610.81	8292.52	69.29	21
Ar^{NesCre}	13563.62	13754.34	6497.94	9647.11	8232.2	181.85	204.03
Ar^{NesCre}	10418.49	8838.58	4255.37	7923.42	8046.16	81.55	27.18
Ar^{NesCre}	13151.12	10579.61	4759.03	10295.95	8428.01	95.38	29.73
Ar^{NesCre}	10687.8	9545.96	4751.24	7662.37	6781.45	114.31	39.76
Ar^{NesCre}	9986.85	7382.68	3603.65	8458.63	6499.23	65.93	46.15
WT	8775.44	6416.94	3037.36	7670.72	11552.21	65.91	17.24
WT	9226.51	6759.56	3549.41	7624.8	11862.07	61.03	25.34
WT	14223.92	15218.76	7452.94	9202.64	8330.83	186.82	77.46
WT	9674.97	10139.29	4234.38	8282.01	5506.2	196.83	75.7
WT	10873.61	10038.17	4932.57	8344.05	7805.6	102.01	33.57
WT	11488.75	11479.26	5227.45	9323.51	5544.52	147.69	42

B

P7.5

	Shank1	*Shank2*	*Shank3*	*Mecp2*	*Psd95*	*Er1*	*Er2*
Ar^{NesCre}	17869.31	13632.48	9186.02	5526.54	24192.58	341.08	20.43
Ar^{NesCre}	16697.62	12374.08	9071.58	5418.45	23992.67	302.95	18.7
Ar^{NesCre}	16533.77	15153.64	9153.78	5980.18	15199.95	559.23	33.58
Ar^{NesCre}	17674.96	15396.63	9863.64	6567.14	15592.19	458.86	21.31
Ar^{NesCre}	18875.45	16427.47	11079.54	6737.62	17683.93	453.67	25.3
Ar^{NesCre}	16840.48	14850.42	8296.77	5556.45	19396.67	459.97	24.55
Ar^{NesCre}	15215.63	12066.9	7650.36	4717.06	23299.25	293.27	16.9
WT	22330.37	21856.25	12099.64	8397.66	15508.04	755.28	30.32
WT	20013.65	24517.29	12513.66	9298.72	13688.52	869.76	18.21
WT	19589.12	18734.03	12478.03	7104.01	16647.28	528.06	24.07
WT	17820.52	15169.95	10618.97	6534.75	17790.36	408.02	27.78
WT	17757.93	16876.46	10995.64	7277.84	13308.28	556.21	47.16
WT	20073.54	18600.38	11790.26	7720.11	17127.21	652.67	19.89
WT	16649.47	13921.2	8302.35	5428.55	22463.43	446.98	19.99

Supplementary Table S4. The absolute numbers of gene expression counts from the nCounter. (A) At E17.5 (B) At P7.5.
